# Delayed Breastfeeding Initiation and Associated Factors in Tanzania: A Cross‐Sectional Study

**DOI:** 10.1002/hsr2.72246

**Published:** 2026-03-30

**Authors:** Linus Paul Rweyemamu, Erneus Ernest, Christopher Hariri Mbotwa

**Affiliations:** ^1^ Department of Molecular Biology and Biotechnology University of Dar es Salaam Dar es Salaam Tanzania; ^2^ Department of Pediatrics and Child Health, Mbeya College of Health and Allied Sciences University of Dar es Salaam Mbeya Tanzania; ^3^ Department of Social Sciences, Mbeya College of Health and Allied Sciences University of Dar es Salaam Mbeya Tanzania

**Keywords:** breastfeeding initiation, demographic and health survey, determinants of health, infant feeding practices, sub‐Saharan Africa, Tanzania

## Abstract

**Background and Aims:**

Delayed initiation of breastfeeding is associated with poor child development outcomes and increased risk of infant mortality, which remains a major public health challenge in low‐ and middle‐income countries. Despite global and national efforts to promote optimal breastfeeding, early initiation remains suboptimal in much of Sub‐Saharan Africa. This study examined the prevalence and factors associated with delayed initiation of breastfeeding in Tanzania.

**Methods:**

We analyzed secondary data from the 2022 Tanzania Demographic and Health Survey and Malaria Indicator Survey, a nationally representative cross‐sectional survey. The study population comprised children under 24 months. The outcome variable was delayed breastfeeding initiation, defined as not initiating breastfeeding within 1 h of life. Weighted data were analyzed using multilevel mixed‐effects logistic regression to identify associated factors.

**Results:**

A total of 4478 children were included, with a mean (±SD) age of 11.5 ± 6.9 months. The prevalence of delayed initiation was 24.4%. Child‐related factors included first (aPR = 1.32; 95% CI: 1.13—1.55) and second birth order (aPR = 1.21; 95% CI: 1.06–1.39). Maternal age 35–49 years was also associated (aPR = 1.16; 95% CI: 1.02–1.32). Health‐related factors included absence of immediate skin‐to‐skin contact (aPR = 1.85; 95% CI: 1.64–2.09), cesarean delivery (aPR = 2.45; 95% CI: 2.17–2.78), lack of birth assistance (aPR = 1.63; 95% CI: 1.16–2.28), and assistance from non‐skilled provider (aPR = 1.42; 95% CI: 1.09–1.84). Geographically, delayed initiation was more prevalent in Zanzibar (aPR = 1.62; 95% CI: 1.33–1.98) and less prevalent in the Southern zone (aPR = 0.54; 95% CI: 0.36–0.80).

**Conclusion:**

Nearly one in four newborns in Tanzania experience delayed breastfeeding initiation. Delayed initiation was associated with child, maternal, health‐related, and geographical factors. Strengthening skin‐to‐skin contact, skilled birth attendance, cesarean support, counseling for first‐time and older mothers, and addressing geographical disparities may improve early initiation and support progress toward Sustainable Development Goal target 3.2.

**Abbreviations**
ANC: antenatal care
aPR: adjusted prevalence ratio
CI: confidence interval
DHS: Demographic and Health Survey
PR: prevalence ratio
SDG: sustainable development goal
SSA: Sub‐Saharan Africa
TDHS‐MIS: Tanzania Demographic and Health Survey and Malaria Indicator Survey
UNICEF: United Nations Children's Fund
WHO: World Health Organization

## 1 Introduction

Breastfeeding is a fundamental component of infant care and is widely recognized as one of the most effective interventions for enhancing infant survival and reducing morbidity and mortality rates [1, 2]. To optimize health and developmental outcomes for both infants and mothers, it is essential that women receive adequate support to initiate breastfeeding as early as possible after birth [3, 4]. In alignment with this, the World Health Organization (WHO) and the United Nations Children's Fund (UNICEF) recommend initiating breastfeeding within the first hour of life, commonly referred to as the “golden hour”, followed by exclusively breastfeeding for the first 6 months, and continuing breastfeeding alongside the gradual introduction of complementary foods for at least 2 years [5].

Delayed initiation of breastfeeding, defined as not providing breast milk to the infant within 1 h after birth, has been shown to significantly increase the risk of neonatal and infant mortality, which remain persistent public health challenges in many low‐ and middle‐income countries, particularly in sub‐Saharan Africa (SSA) [2, 3, 6]. Early initiation ensures that the infant receives colostrum, which is rich in oligosaccharides and other essential nutrients vital for healthy growth and cognitive development [7]. It also provides antibodies that protect against infections such as pneumonia, diarrhea, and neonatal sepsis, while supporting immune system development [8, 9, 10]. Furthermore, early initiation is a strong predictor of exclusive breastfeeding and has been associated with enhanced mother‐infant bonding through skin‐to‐skin contact, as well as reduced risk of obesity, hypoglycemia, and other non‐communicable diseases later in life [11, 12, 13]. For mothers, early initiation offers both immediate and long‐term health benefits, including reduced postpartum obesity, improved birth spacing, and a lower risk of breast and ovarian cancers [10].

Despite these well‐established benefits, a significant proportion of women, particularly in SSA, experience delays in initiating breastfeeding, thereby missing these critical advantages for both mother and child [3, 13, 14, 15, 16, 17, 18, 19]. Globally, nearly half of all newborns are breastfed more than 1 h after birth, indicating a widespread gap in meeting WHO and UNICEF recommendations on infant and young child feeding [20]. A pooled analysis from SSA estimated the prevalence of delayed initiation at 47%, with country‐specific rates, ranging from as low as 4% in Malawi to as high as 83% in Guinea [21]. In Tanzania, although nationally representative data have been limited, previous regional studies reported varying prevalence rates ranging from 17% to 51% [22, 23, 24, 25].

Various studies conducted across SSA have identified a range of factors associated with initiation of breastfeeding [14, 15, 16, 17, 18, 22, 24, 26]. These include newborn characteristics such as birth weight [16, 17], sex of the child [24, 27], and birth order [26]; maternal demographic and obstetric factors such as age, educational level, occupation, number of antenatal care visits, and skin‐to‐skin contact [14, 15, 22]; and household and community‐level factors such as place and mode of delivery [14, 18, 24], assistance during delivery [26], household wealth index [15, 18], media exposure [26], place of residence [15, 16], and geographic region [15]. However, to the best of our knowledge, no published study has investigated the factors associated with delayed initiation of breastfeeding in Tanzania using the most recent nationally representative data.

Therefore, this study aimed to examine the prevalence and factors associated with delayed breastfeeding initiation using data from the 2022 Tanzania Demographic and Health Survey and Malaria Indicator Survey (2022 TDHS‐MIS). Understanding the prevalence and factors associated with delayed breastfeeding initiation in the Tanzanian context is crucial for guiding the development of targeted, evidence‐based interventions to improve maternal and child health outcomes. Such efforts are also vital for accelerating progress toward Sustainable Development Goal (SDG) 3.2, which seeks to end preventable deaths of newborns and children under‐5 by 2030 [28].

## 2 Methods

### 2.1 Study Design

This study involved a secondary analysis of data from the 2022 TDHS‐MIS, a nationally representative cross‐sectional survey that collected information on maternal and child health, reproductive health, and fertility. Detailed methodology and survey procedures are available in the TDHS‐MIS final report [29]. The reporting of this study conforms to STROBE guidelines [30].

### 2.2 Study Population and Sample Size

The study population comprised all children aged below 24 months. The 2022 TDHS‐MIS employed a stratified two‐stage sampling design covering Tanzania mainland (urban/rural) and Zanzibar. In the first stage, 629 clusters were selected with probability proportional to size. In the second stage, 26 households were systematically selected per cluster, yielding 16,312 households. Of these, 15,705 were successfully interviewed (99% response), identifying 15,699 eligible women aged 15–49, of whom 15,254 completed interviews (97% response). Among these, 4308 women had at least one child aged < 24 months, resulting in a total of 4478 children included in the analysis.

### 2.3 Study Variables

#### 2.3.1 Outcome Variable

The outcome variable was delayed initiation of breastfeeding, defined as not initiating breastfeeding within 1 h after birth. It was based on maternal report and coded as “1” if breastfeeding was initiated after 1 h and “0” otherwise. This definition follows WHO recommendations and has been widely applied in similar studies [5, 14, 16, 18, 31].

#### 2.3.2 Independent Variables

Several independent variables were included in the analysis based on prior literature [14, 16, 18] and their availability in the 2022 TDHS‐MIS dataset. These variables were categorized into four domains: *child characteristics* (sex, birth weight, birth order, twin status); *maternal characteristics* (age, education, and marital status); *healthcare‐related factors* (antenatal care [ANC] visits, skin‐to‐skin contact, place of delivery, assistance during delivery, mode of delivery, and presence of a birth companion); and *socioeconomic factors* (exposure to mass media exposure, place of residence, telephone ownership, internet use, geographical zones, and wealth index status constructed using principal component analysis based on household assets, housing characteristics, and access to utilities). Detailed definitions and coding of these variables are provided in the Guide to DHS Statistics [32].

### 2.4 Ethical Approval and Consent to Participate

Formal ethical approval was not required for this study because the dataset is publicly available. However, permission to use the data was obtained from the DHS Program. The 2022 TDHS‐MIS itself was conducted with approval from national and international review boards, including the National Institute of Medical Research, the Zanzibar Medical Research Ethical Committee, the Institutional Review Board of ICF, and the Centers for Disease Control and Prevention in Atlanta. Informed consent was obtained from all participants before the commencement of the survey.

### 2.5 Statistical Analyses

Data were extracted from the DHS Under‐5 Children Recode file (TZKR81FL). Continuous variables were summarized using means and standard deviations, whereas categorical variables were described using frequencies and percentages. All analyses incorporated sampling weights to account for the complex DHS survey design, including stratification, clustering, and unequal probabilities of selection.

Associations between delayed breastfeeding initiation and independent variables were examined using mixed‐effects generalized linear models with a Poisson family and robust standard errors to account for correlation among children born to the same mother. Bivariate analyses were first conducted to estimate crude prevalence ratios (PRs). Variables with *p*‐values < 0.20 in the bivariate analysis were considered for inclusion in the multivariable model to estimate adjusted prevalence ratios (aPRs). The use of a 0.20 threshold at the screening stage has been shown to enhance model performance by reducing the risk of excluding potentially important covariates [33, 34]. Additionally, careful variable selection was undertaken to avoid overparameterization, as including an excessive number of variables relative to the sample size and number of clusters may result in model instability, overfitting, and convergence difficulties [35].

All statistical tests were two‐tailed, and statistical significance was defined at *p* < 0.05. Missing data were assessed for all study variables. Birth weight and number of ANC visits had missing values. In descriptive analyses, missing values were excluded from the numerators but retained in the denominators when calculating proportions, in accordance with DHS statistical guidelines [32]. For regression analyses, a complete‐case approach was applied, whereby observations with missing data on variables included in the model were excluded. All analyses were performed using Stata version 18.

## 3 Results

### 3.1 Characteristics of the Study Participants

A total of 4478 children were included in the analysis, with a mean age of 11.5 ± 6.9 months. Just over half were male. Most children were born with normal birth weight, were of third or higher birth order, and were singletons. The mean maternal age was 28.2 ± 7.0 years. The majority of mothers lived in rural areas, had at least primary education, and were in a marital union. Regarding maternal healthcare factors, nearly two‐thirds reported attending four or more ANC visits during their most recent pregnancy. Most deliveries were spontaneous vaginal births, occurred in health facilities, and were attended by skilled personnel. About half of the newborns experienced immediate skin‐to‐skin contact (Table 1).

**Table 1:** Child, maternal, health‐related, and socioeconomic characteristics.

Variable	Count (%)
*Child characteristics*	
Sex of child	
Male	2297 (51.3)
Female	2181 (48.7)
Birth weight	
Low birth weight (< 2500 g)	320 (7.1)
Normal birth weight (2500–3999 g)	3077 (68.7)
Macrosomia (4000+ g)	127 (2.8)
Unknown (undetermined at birth)	954 (21.3)
Birth order	
1	1019 (22.8)
2	916 (20.5)
3+	2543 (56.8)
Twin status	
Singleton	4328 (96.7)
Multiple	150 (3.3)
*Maternal characteristics*	
Age	
15–24	1570 (35.1)
25–34	1963 (43.8)
35–49	945 (21.1)
Educational level	
No formal education	899 (20.1)
Primary	2262 (50.5)
Secondary or higher	1317 (29.4)
Marital status	
Never in union	349 (7.8)
In marital union	3746 (83.7)
Previously in union	383 (8.6)
*Health‐related characteristics*	
Number of antenatal care visits	
No visit	457 (10.2)
1–3	1065 (23.8)
4–7	2652 (59.2)
8–10	121 (2.7)
Unknown/missing	183 (4.1)
Skin‐to‐skin contact	
No	2142 (47.8)
Yes	2336 (52.2)
Delivery in health facility	
No	839 (18.7)
Yes	3639 (81.3)
Type of assistance during delivery	
None	104 (2.3)
Non‐skilled provider	576 (12.9)
Skilled provider	3798 (84.8)
Delivery by caesarean section	
No	3979 (88.9)
Yes	499 (11.1)
Presence of birth companion at delivery	
No	1199 (26.8)
Yes	3279 (73.2)
*Socioeconomic characteristics*	
Exposure to mass media	
Newspaper	883 (19.7)
Radio	2415 (53.9)
Television	1794 (40.1)
Place of residence	
Urban	1229 (27.4)
Rural	3249 (72.6)
Household wealth index	
Poor	1828 (40.8)
Middle	932 (20.8)
Rich	1718 (38.4)
Telephone ownership	
No telephone	1933 (43.2)
Non‐smartphone	1936 (43.2)
Smartphone	609 (13.6)
Internet use in last 12 months	
No	3962 (88.5)
Yes	516 (11.5)
Geographical zone	
Western	406 (9.1)
Northern	381 (8.5)
Central	380 (8.5)
Southern highlands	320 (7.1)
Southern	176 (3.9)
Southwest highlands	585 (13.1)
Lake	1116 (24.9)
Eastern	421 (9.4)
Zanzibar	693 (15.5)

### 3.2 Prevalence of Delayed Breastfeeding Initiation

**Figure 1:**
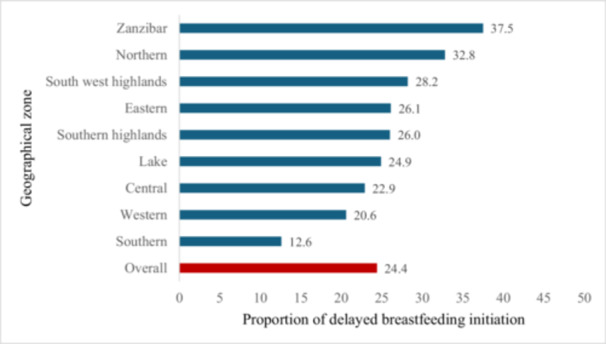
Prevalence of delayed breastfeeding initiation by geographical zones in Tanzania. Delayed breastfeeding initiation was defined as initiation of breastfeeding more than 1 h after birth.

Overall, 95.4% of children included in this study had ever been breastfed. The weighted prevalence of delayed breastfeeding initiation was 24.4% (95% CI: 22.77–26.04). The prevalence varied across zones, ranging from 12.6% in the Southern zone to 37.5% in Zanzibar (Figure 1).

### 3.3 Factors Associated With Delayed Breastfeeding Initiation

Table 2 presents the results of the generalized mixed‐effects Poisson regression analysis examining factors associated with delayed breastfeeding initiation among women aged 15–49 years in Tanzania. In the adjusted model, higher prevalence of delayed initiation was observed among first‐ and second‐born children and among mothers aged 35–49 years. Health‐related factors demonstrated the strongest associations, with absence of immediate skin‐to‐skin contact, lack of delivery assistance, assistance by non‐skilled personnel, and caesarean delivery independently associated with increased prevalence of delayed initiation. Geographic disparities were also observed, with higher prevalence in Zanzibar and lower prevalence in the Southern zone compared with the Eastern zone.

**Table 2:** Weighted bivariate and multivariable analyses for the factors associated with delayed breastfeeding initiation.

Variable	*N*	Delayed breastfeeding initiation, weighted (%)	Bivariate analysis		Multivariable analysis	
			PR (95% CI)	*p*‐value	aPR (95% CI)	*p*‐value
Overall	4478	24.37				
*Child factors*						
Sex						
Male (Ref.)	2297	25.77	Ref.			
Female	2181	25.28	0.98 (0.86–1.11)	0.77		
Birth weight						
Low	320	25.77	1.08 (0.85–1.38)	0.53		
Normal (Ref.)	3077	24.26	Ref.			
Macrosomia	127	22.63	0.95 (0.65–1.39)	0.78		
Birth order						
1	1019	31.21	1.35 (1.17–1.57)	< 0.001	1.32 (1.13–1.55)	0.001
2	916	26.00	1.13 (0.96–1.32)	0.14	1.21 (1.06–1.39)	0.004
3+ (Ref.)	2543	23.07	Ref.		Ref.	
Twin status						
Single (Ref.)	4328	25.32	Ref.			
Multiple	150	31.90	1.22 (0.78–1.89)	0.38	1.11 (0.82–1.51)	0.51
*Maternal factors*						
Age						
15—24	1570	27.46	1.18 (1.02–1.36)	0.03	1.05 (0.92–1.20)	0.47
25—34 (Ref.)	1963	23.34	Ref.		Ref.	
35—49	945	26.69	1.14 (0.97–1.35)	0.11	1.16 (1.02–1.32)	0.02
Educational level						
No formal education	899	22.18	0.71 (0.59–0.86)	< 0.001	0.88 (0.74–1.05)	0.17
Primary	2262	24.30	0.78 (0.68–0.90)	0.001	0.98 (0.87–1.12)	0.81
Secondary or higher (Ref.)	1317	31.11	Ref.		Ref.	
Marital status						
Never in union	349	29.37	1.16 (0.94–1.44)	0.16	1.02 (0.86–1.21)	0.84
In a marital union (Ref.)	3746	25.21	Ref.			
Previously in a union	383	24.80	0.98 (0.78–1.23)	0.89		
*Health‐related factors*						
Number of ANC visits						
No visit	457	25.62	1.01 (0.83–1.24)	0.89		
1–3	1065	25.87	1.02 (0.88–1.19)	0.74		
4+ (Ref.)	2773	25.24	Ref.			
Skin‐to‐skin contact						
No	2142	37.59	2.55 (2.21–2.94)	< 0.001	1.85 (1.64–2.09)	< 0.001
Yes (Ref.)	2336	14.74	Ref.		Ref.	
Delivery in health facility						
No	839	34.50	1.47 (1.28–1.68)	< 0.001	1.20 (0.93–1.55)	0.16
Yes (Ref.)	3639	23.52	Ref.		Ref.	
Type of assistance during delivery						
None	104	39.31	1.67 (1.26–2.20)	< 0.001	1.63 (1.16–2.28)	0.005
Non‐skilled provider	576	36.42	1.54 (1.32–1.80)	< 0.001	1.42 (1.09–1.84)	0.009
Skilled provider (Ref.)	3798	23.60	Ref.		Ref.	
Delivery by caesarean section						
No (Ref.)	3979	20.92	Ref.		Ref.	
Yes	499	66.08	3.16 (2.83–3.52)	< 0.001	2.45 (2.17–2.78)	< 0.001
Presence of birth companion at delivery						
No	1199	27.35	1.09 (0.94–1.27)	0.23	1.09 (0.98–1.22)	0.11
Yes (Ref.)	3279	25.03	Ref.		Ref.	
*Socioeconomic factors*						
Exposure to mass media						
Newspaper	883	29.16	1.18 (1.01–1.37)	0.04	0.94 (0.83–1.06)	0.32
Radio	2415	26.98	1.13 (1.00–1.28)	0.06	1.01 (0.90–1.12)	0.90
Television	1794	28.81	1.23 (1.09–1.40)	0.001	1.06 (0.93–1.19)	0.40
Place of residence						
Urban (Ref.)	1229	30.32	Ref.		Ref.	
Rural	3249	23.71	0.78 (0.68–0.90)	< 0.001	0.97 (0.85–1.10)	0.62
Household wealth index						
Poor	1828	23.94	1.07 (0.91–1.27)	0.42		
Middle (Ref.)	932	22.33	Ref.		Ref.	
Rich	1718	28.93	1.30 (1.09–1.54)	0.004	1.01 (0.87–1.16)	0.93
Mobile phone ownership						
No phone	1933	23.46	0.63 (0.53–0.75)	< 0.001	1.13 (0.91–1.40)	0.27
Non‐smartphone	1936	24.39	0.65 (0.55–0.78)	< 0.001	0.97 (0.86–1.09)	0.58
Smartphone (Ref.)	609	37.49	Ref.		Ref.	
Used internet in the last 12 months	516	38.45	0.63 (0.53–0.74)	< 0.001	0.96 (0.78–1.19)	0.73
Geographical zone						
Western	406	20.63	0.79 (0.60–1.03)	0.09	1.00 (0.77–1.30)	0.99
Northern	381	32.82	1.26 (0.99–1.60)	0.06	1.21 (1.00–1.48)	0.06
Central	380	22.91	0.88 (0.66–1.17)	0.37	0.80 (0.62–1.02)	0.07
Southern Highlands	320	25.95	0.99 (0.76–1.30)	0.97	0.97 (0.77–1.21)	0.79
Southern	176	12.62	0.48 (0.31–0.75)	0.001	0.54 (0.36–0.80)	0.002
Southwest Highlands	585	28.18	1.08 (0.85–1.37)	0.53	1.10 (0.90–1.34)	0.35
Lake	1116	24.89	0.95 (0.76–1.19)	0.67	1.05 (0.86–1.27)	0.63
Eastern (Ref.)	421	26.11	Ref.		Ref.	
Zanzibar	693	37.53	1.44 (1.15–1.79)	0.001	1.62 (1.33–1.98)	< 0.001

*Note:* In the multivariable analysis, higher prevalence of delayed breastfeeding initiation was observed among first‐ and second‐born children and among mothers aged 35–49 years. Absence of skin‐to‐skin contact, delivery without skilled assistance, and caesarean section were strongly associated with delayed initiation. Geographical differences were evident, with lower prevalence in the Southern zone and higher prevalence in Zanzibar. No other factors remained significant after adjustment.

Abbreviations: ANC, antenatal care; aPR, adjusted prevalence ratio; CI, confidence interval; PR, prevalence ratio; Ref, reference category.

## 4 Discussion

The United Nations SDG 3.2 calls for accelerated efforts to end preventable deaths among newborns and children under‐5 by 2030, with specific targets of reducing neonatal mortality to at least 12 per 1000 live births and under‐5 mortality to at least 25 per 1000 live births [28]. Early initiation of breastfeeding is a key strategy endorsed by WHO and UNICEF to achieve these targets, given its well‐documented benefits for both maternal and child health [7, 8, 9, 10]. In this context, understanding the prevalence and factors associated with delayed initiation provides valuable insights for guiding maternal and child health programs, particularly in low‐ and middle‐income countries. Using nationally representative data, this study examined the prevalence of delayed initiation of breastfeeding in Tanzania and factors associated with it.

The findings indicate that approximately one in four newborns (24.4%) were not breastfed within the first hour of birth. Factors associated with delayed initiation clustered into four broad categories: child characteristics (first‐ and second‐birth order), maternal characteristics (age 35–49 years), health‐related factors (absence of skin‐to‐skin contact, cesarean delivery, and non‐skilled or no delivery assistance), and socioeconomic factors (geographic disparities, with higher prevalence in Zanzibar and lower prevalence in the Southern zone).

This prevalence estimate, derived from nationally representative dataset, differs from earlier sub‐national reports in Tanzania, which reported delayed initiation ranging from 16% in a northern municipality [23] to around 30% in other areas of the same region [25, 36], 38% in Simiyu [24], and up to 49% in selected rural districts [22]. At the national level, survey data suggest a decline over time, with delayed initiation reported at 53% in the 2010 TDHS [37], 49% in the 2015/16 TDHS [38], and 24% in the 2022 TDHS‐MIS (analyzed in this study). This downward trend indicates progress Tanzania is making toward achieving global targets under the SDG 3.2 [28]. Similar prevalence levels have been observed in Ethiopia (26%) [14] and Mozambique (25%) [16], suggesting that delayed initiation remains a widespread challenge across SSA. These similarities point to shared systemic barriers, such as limited access to skilled care at birth, and challenges in implementing of breastfeeding promotion strategies. Collectively, these findings suggest that delayed breastfeeding initiation is not merely a country‐specific issue but a regional concern, suggesting the need for context‐specific and coordinated strategies to strengthen early breastfeeding practices across SSA.

Child‐related factors were notable, with first‐ and second‐order births showing higher prevalence of delayed initiation compared to higher‐order births. Finding from Ghana [26], Mauritania [18], and Zimbabwe [17] also demonstrated this pattern. One possible explanation is that first‐time mothers may face challenges such as lack of confidence, delivery‐related fatigue, or anxiety, whereas multiparous women may benefit from greater familiarity with newborn care and breastfeeding practices.

Maternal age was also associated with delayed initiation. Women aged 35 to 49 years had a higher prevalence compared with those aged 25 to 34 years. Although this pattern differs from findings in Ethiopia [14], where younger mothers showed higher prevalence, it is partly consistent with results from The Gambia [15]. These differences across settings may reflect variations in health‐seeking behaviors, maternal health conditions, and levels of support during childbirth. These findings suggest the importance of ensuring that older mothers receive adequate support and counseling during delivery and the immediate postpartum period.

Health‐related factors played an important role. The absence of skin‐to‐skin contact was associated with delayed initiation, consistent with studies from Mauritania [18], Zimbabwe [17], and Papua New Guinea [31]. Skin‐to‐skin contact has been shown to stimulate neonatal reflexes, stabilize infant physiology, and support maternal oxytocin release, all of which are linked to timely breastfeeding initiation [17]. Ensuring uninterrupted skin‐to‐skin contact in health facilities may therefore be a key practice for improving early initiation [39].

The type of delivery assistance was another important factor. Women delivering without assistance or with non‐skilled attendants showed higher prevalence of delayed initiation compared with those assisted by skilled professionals. Similar associations have been observed in Ghana [26], Ethiopia [40], and Zimbabwe [17]. These results underscore the role of skilled birth attendance in facilitating breastfeeding initiation, although systemic barriers such as reliance on traditional birth attendants and limited access to skilled services must also be considered. Strengthening health systems and exploring ways to integrate traditional attendants into formal care structures may help reduce delays.

Mode of delivery also showed a strong association, with cesarean birth linked to delayed initiation compared with vaginal delivery. This association has been consistently reported in Tanzania [22, 23], across SSA countries [14, 16, 17, 18, 40], and Asian countries such as Bangladesh [41] and Papua New Guinea [31]. Factors contributing to this pattern may include delayed maternal recovery, temporary mother‐infant separation, and post‐operative care routines. Global recommendations highlight the importance of minimizing separation and promoting skin‐to‐skin contact even in surgical context [39]. Strengthening adherence to these recommendations may improve initiation practices following cesarean delivery.

Geographic disparities were also evident. Women in Zanzibar reported higher prevalence of delayed initiation, while those in the Southern zone had a lower prevalence compared to their counterparts in the Eastern zone. Similar geographical variations have been observed in The Gambia [15], Ghana [26], Mauritania [18], and Ethiopia [40]. These differences may be influenced by sociocultural norms, health system capacity, and the extent of breastfeeding promotion programs. Tailored interventions, including strengthening facility‐level support in Zanzibar and reinforcing community‐based strategies aligned with the Baby‐Friendly Hospital Initiative [39], may help address these disparities.

### 4.1 Strengths and Limitations

A key strength of this study is the use of a large, nationally representative dataset collected with rigorous methodology and analyzed using mixed‐effects modeling, which supports the robustness and generalizability of the findings. However, several limitations must be considered. First, the cross‐sectional design of the 2022 TDHS‐MIS limits the ability to draw causal inferences between predictors and delayed breastfeeding initiation. Second, the reliance on women's self‐reported information introduces the possibility of recall and social desirability biases, particularly for events surrounding childbirth. Third, the analysis was restricted to variables available in the 2022 TDHS‐MIS, excluding other potentially relevant factors such as maternal knowledge and attitudes, cultural practices, quality of antenatal care, and broader health system factors. Despite these limitations, the study provides nationally representative insights into breastfeeding initiation in Tanzania and contributes evidence relevant to similar settings in SSA.

## 5 Conclusion

This study found that nearly one in four newborns in Tanzania are not breastfed within the first hour of life, reflecting suboptimal adherence to WHO recommendations. Delayed initiation was associated with first‐ and second‐order births, advanced maternal age, absence of immediate skin‐to‐skin contact, cesarean delivery, lack of skilled birth assistance, and regional disparities, with higher prevalence in Zanzibar and lower prevalence in the Southern zone. These findings point to the need for targeted efforts to promote early breastfeeding practices. Potential approaches include encouraging immediate skin‐to‐skin contact, strengthening skilled birth attendance, providing dedicated support for mothers undergoing cesarean delivery, and addressing regional disparities through community education and health system support. Integrating early breastfeeding counseling into antenatal and postnatal services, particularly for first‐time and older mothers, may help strengthen early initiation practices and contribute to national progress toward improved child health and SDG target 3.2.

## Author Declaration

All authors have read and approved the final version of the manuscript. Linus Paul Rweyemamu had full access to all of the data in this study and takes complete responsibility for the integrity of the data and the accuracy of the data analysis.

## Author Contributions

**Linus Paul Rweyemamu:** conceptualization, investigation, writing – original draft, methodology, writing – review and editing, formal analysis, project administration, supervision, resources, data curation. **Erneus Ernest:** conceptualization, investigation, data curation, formal analysis, methodology, validation, and writing – review and editing. **Christopher Hariri Mbotwa:** conceptualization, investigation, methodology, writing – original draft, data curation, formal analysis, validation, writing – review and editing, software.

## Funding

The authors received no specific funding for this work.

## Conflicts of Interest

The authors declare no conflicts of interest.

## Transparency Statement

The lead author Linus Paul Rweyemamu affirms that this manuscript is an honest, accurate, and transparent account of the study being reported; that no important aspects of the study have been omitted; and that any discrepancies from the study as planned (and, if relevant, registered) have been explained.

## Data Availability

The data set used in this study is publicly available on the DHS website (https://dhsprogram.com/Data/).

## References

[1] N. C. Rollins , N. Bhandari , N. Hajeebhoy , et al., “Why Invest, and What It Will Take to Improve Breastfeeding Practices?,” Lancet 387, no. 10017 (2016): 491–504. 26869576 10.1016/S0140-6736(15)01044-2

[2] E. R. Smith , L. Hurt , R. Chowdhury , B. Sinha , W. Fawzi , and K. M. Edmond , “Delayed Breastfeeding Initiation and Infant Survival: A Systematic Review and Meta‐Analysis,” PLoS One 12, no. 7 (2017): e0180722. 28746353 10.1371/journal.pone.0180722 PMC5528898

[3] M. Ekholuenetale and A. Barrow , “What Does Early Initiation and Duration of Breastfeeding Have to Do With Childhood Mortality? Analysis of Pooled Population‐Based Data in 35 Sub‐Saharan African Countries,” International Breastfeeding Journal 16 (2021): 91. 34876163 10.1186/s13006-021-00440-x PMC8650286

[4] WHO, *Guideline: Protecting, Promoting and Supporting Breastfeeding in Facilities Providing Maternity and Newborn Services* (WHO, 2017). 29565522

[5] WHO and UNICEF , *Indicators for Assessing Infant and Young Child Feeding Practices: Definitions and Measurement Methods* (WHO and UNICEF, 2021).

[6] E. R. Smith , L. M. Locks , K. P. Manji , et al., “Delayed Breastfeeding Initiation Is Associated With Infant Morbidity,” Journal of Pediatrics 191 (2017): 57–62.e52. 29173323 10.1016/j.jpeds.2017.08.069 PMC8011584

[7] M. Chiurazzi , M. Cozzolino , T. Reinelt , et al., “Human Milk and Brain Development in Infants,” Reproductive Medicine 2, no. 2 (2021): 107–117.

[8] A. K. Debes , A. Kohli , N. Walker , K. Edmond , and L. C. Mullany , “Time to Initiation of Breastfeeding and Neonatal Mortality and Morbidity: A Systematic Review,” BMC Public Health 13 (2013): S19. 24564770 10.1186/1471-2458-13-S3-S19 PMC3847227

[9] B. M. Abie and Y. A. Goshu , “Early Initiation of Breastfeeding and Colostrum Feeding Among Mothers of Children Aged Less Than 24 Months in Debre Tabor, Northwest Ethiopia: A Cross‐Sectional Study,” BMC Research Notes 12 (2019): 65. 30696481 10.1186/s13104-019-4094-6 PMC6352422

[10] P. K. Osei and A. K. Anderson , *Infant Nutrition and Feeding in the First 2 Years of Life* (2023).

[11] M. Gayatri and G. L. Dasvarma , “Predictors of Early Initiation of Breastfeeding in Indonesia: A Population‐Based Cross‐Sectional Survey,” PLoS One 15, no. 9 (2020): e0239446. 32970729 10.1371/journal.pone.0239446 PMC7514028

[12] T. A. E. Permatasari and A. Syafruddin , “Early Initiation of Breastfeeding Related to Exclusive Breastfeeding and Breastfeeding Duration in Rural and Urban Areas in Subang, West Java, Indonesia,” Journal of Health Research 30, no. 5 (2016): 337–345.

[13] R. G. Aboagye , B. O. Ahinkorah , A.‐A. Seidu , S. K. Anin , J. B. Frimpong , and J. E. Hagan , “Mother and Newborn Skin‐To‐Skin Contact and Timely Initiation of Breastfeeding in Sub‐Saharan Africa,” PLoS One 18, no. 1 (2023): e0280053. 36626377 10.1371/journal.pone.0280053 PMC9831337

[14] R. N. Haile , B. B. Abate , and T. A. Kitaw , “Predictors of Late Initiation of Breastfeeding Practice in Ethiopia: A Multilevel Mixed‐Effects Analysis of Recent Evidence From EDHS 2019,” BMJ Open 14, no. 4 (2024): e081069. 10.1136/bmjopen-2023-081069 PMC11015321 38604642

[15] M. L. Darboe , A. Jeyakumar , S. M. A. Mansour , and S. Valawalkar , “Determinants of Early Initiation of Breastfeeding in The Gambia: A Population‐Based Study Using the 2019–2020 Demographic and Health Survey Data,” International Breastfeeding Journal 18, no. 1 (2023): 33. 37349805 10.1186/s13006-023-00570-4 PMC10288753

[16] E. G. Mekonen , “Individual‐And Community‐Level Factors Associated With Early Initiation of Breastfeeding in Mozambique: Evidence From the 2022–2023 Demographic and Health Survey,” International Breastfeeding Journal 19, no. 1 (2024): 81. 39722032 10.1186/s13006-024-00691-4 PMC11670390

[17] F. Mukora‐Mutseyekwa , H. Gunguwo , R. G. Mandigo , and P. Mundagowa , “Predictors of Early Initiation of Breastfeeding Among Zimbabwean Women: Secondary Analysis of ZDHS 2015,” Maternal Health, Neonatology and Perinatology 5 (2019): 2. 30675366 10.1186/s40748-018-0097-x PMC6332660

[18] M. Sarfo , J. Aggrey‐Korsah , L. A. Adzigbli , et al., “Prevalence of Early Initiation of Breastfeeding and Its Associated Factors Among Women in Mauritania: Evidence From a National Survey,” International Breastfeeding Journal 19, no. 1 (2024): 69. 39358717 10.1186/s13006-024-00669-2 PMC11448303

[19] F. Appiah , B. O. Ahinkorah , E. Budu , et al., “Maternal and Child Factors Associated With Timely Initiation of Breastfeeding in Sub‐Saharan Africa,” International Breastfeeding Journal 16 (2021): 55. 34281591 10.1186/s13006-021-00402-3 PMC8287803

[20] K. Takahashi , T. Ganchimeg , E. Ota , et al., “Prevalence of Early Initiation of Breastfeeding and Determinants of Delayed Initiation of Breastfeeding: Secondary Analysis of the WHO Global Survey,” Scientific Reports 7, no. 1 (2017): 44868. 28322265 10.1038/srep44868 PMC5359598

[21] B. Olapeju , M. Bride , M. Wamala , D. Atobrah , E. H. Lee , and Z. M. Hendrickson , “Antenatal Care and Breastfeeding Practices in Sub‐Saharan Africa: An Analysis of Demographic and Health Surveys,” BMC Pregnancy and Childbirth 25, no. 1 (2025): 77. 39871181 10.1186/s12884-025-07188-w PMC11770989

[22] A. Exavery , A. M. Kanté , A. Hingora , and J. F. Phillips , “Determinants of Early Initiation of Breastfeeding in Rural Tanzania,” International Breastfeeding Journal 10 (2015): 27. 26413139 10.1186/s13006-015-0052-7 PMC4582933

[23] H. Y. Lyellu , T. H. Hussein , M. Wandel , B. Stray‐Pedersen , M. Mgongo , and S. E. Msuya , “Prevalence and Factors Associated With Early Initiation of Breastfeeding Among Women in Moshi Municipal, Northern Tanzania,” BMC Pregnancy and Childbirth 20 (2020): 285. 32393191 10.1186/s12884-020-02966-0 PMC7216396

[24] L. J. Shirima , H. L. Mlay , S. Mkuwa , et al., “Early Initiation of Breastfeeding and Associated Factors Among Women of Reproductive Age in Simiyu Region, Tanzania,” SAGE Open Nursing 9 (2023): 23779608231209142. 37942408 10.1177/23779608231209142 PMC10629309

[25] F. Ali , M. Mgongo , R. Mamseri , J. M. George , I. B. Mboya , and S. E. Msuya , “Prevalence of and Factors Associated With Early Initiation of Breastfeeding Among Women With Children Aged 24 Months in Kilimanjaro Region, Northern Tanzania: A Community‐Based Cross‐Sectional Study,” International Breastfeeding Journal 15 (2020): 1–10. 32912320 10.1186/s13006-020-00322-8 PMC7488056

[26] A.‐A. Seidu , E. K. Ameyaw , B. O. Ahinkorah , and F. Bonsu , “Determinants of Early Initiation of Breastfeeding in Ghana: A Population‐Based Cross‐Sectional Study Using the 2014 Demographic and Health Survey Data,” BMC Pregnancy and Childbirth 20 (2020): 632. 33076852 10.1186/s12884-020-03308-w PMC7574209

[27] O. A. Bolarinwa , B. O. Ahinkorah , F. Arthur‐Holmes , et al., “Sex Inequality in Early Initiation of Breastfeeding in 24 Sub‐Saharan African Countries: A Multi‐Country Analysis of Demographic and Health Surveys,” PLoS One 17, no. 5 (2022): e0267703. 35587942 10.1371/journal.pone.0267703 PMC9119560

[28] W.‐K. Chiu and B. Y. F. Fong , “Sustainable Development Goal 3 in Healthcare.” Environmental, Social and Governance and Sustainable Development in Healthcare (Springer, 2023), 33–45.

[29] Ministry of Health (Tanzania Mainland), Ministry of Health (Zanzibar), National Bureau of Statistics, Office of the Chief Government Statistician, and ICF, *Tanzania Demographic And Health Survey and Malaria Indicator Survey 2022 Final Report, Dodoma, Tanzania, and Rockville, Maryland, USA* (2023).

[30] E. Von Elm , D. G. Altman , M. Egger , S. J. Pocock , P. C. Gøtzsche , and J. P. Vandenbroucke , “The Strengthening the Reporting of Observational Studies in Epidemiology (STROBE) Statement: Guidelines for Reporting Observational Studies,” Lancet 370, no. 9596 (2007): 1453–1457. 18064739 10.1016/S0140-6736(07)61602-X

[31] A.‐A. Seidu , B. O. Ahinkorah , E. Agbaglo , et al., “Determinants of Early Initiation of Breastfeeding in Papua New Guinea: A Population‐Based Study Using the 2016–2018 Demographic and Health Survey Data,” Archives of Public Health 78 (2020): 124. 33292575 10.1186/s13690-020-00506-y PMC7684736

[32] T. N. Croft , A. M. J. Marshall , C. K. Allen , et al., *Guide to DHS Statistics* (2018).

[33] D. Zellner , F. Keller , and G. E. Zellner , “Variable Selection in Logistic Regression Models,” Communications in Statistics‐Simulation and Computation 33, no. 3 (2004): 787–805.

[34] J. D. W. Hosmer , S. Lemeshow , and R. X. Sturdivant , Applied Logistic Regression (John Wiley & Sons, 2013).

[35] J. Lever , M. Krzywinski , and N. Altman , “Model Selection and Overfitting,” Nature Methods 13, no. 9 (2016): 703–704.

[36] F. Kiwango , I. B. Mboya , B. John , T. Hashim , S. E. Msuya , and M. Mgongo , “Prevalence and Factors Associated With Timely Initiation of Breastfeeding in Kilimanjaro Region, Northern Tanzania: A Cross‐Sectional Study,” BMC Pregnancy and Childbirth 20, no. 1 (2020): 505. 32873243 10.1186/s12884-020-03209-y PMC7465800

[37] R. Victor , S. K. Baines , K. E. Agho , and M. J. Dibley , “Determinants of Breastfeeding Indicators Among Children Less Than 24 Months of Age in Tanzania: A Secondary Analysis of the 2010 Tanzania Demographic and Health Survey,” BMJ Open 3, no. 1 (2013): e001529. 10.1136/bmjopen-2012-001529 PMC3549262 23299109

[38] H. P. Maro , T. Mtuy , L. J. Msuya , B. Moshi , and M. J. Mahande , “Trends and Factors Associated With Changes in Early Initiation of Breastfeeding: Analysis of the Tanzania Demographic and Health Surveys 2004‐2016,” Open Journal of Epidemiology 12, no. 4 (2022): 505–520.

[39] WHO , *Implementation Guidance: Protecting, Promoting and Supporting Breastfeeding in Facilities Providing Maternity and Newborn Services—The Revised Baby‐Friendly Hospital Initiative* (World Health Organization, 2018). 29565522

[40] J. R. John , S. K. Mistry , G. Kebede , N. Manohar , and A. Arora , “Determinants of Early Initiation of Breastfeeding in Ethiopia: A Population‐Based Study Using the 2016 Demographic and Health Survey Data,” BMC Pregnancy and Childbirth 19, no. 1 (2019): 69. 30760226 10.1186/s12884-019-2211-0 PMC6373137

[41] S. Raihana , A. Alam , T. M. Huda , and M. J. Dibley , “Factors Associated With Delayed Initiation of Breastfeeding in Health Facilities: Secondary Analysis of Bangladesh Demographic and Health Survey 2014,” International Breastfeeding Journal 16, no. 1 (2021): 14. 33482847 10.1186/s13006-021-00360-w PMC7821485

